# Prevalence and associated factors of common mental disorders among medical students at a university in Brazil

**DOI:** 10.1192/j.eurpsy.2021.988

**Published:** 2021-08-13

**Authors:** T. Prata, E. Vasconcelhos, D. Calcides, A. Carvalho, E. De Melo, E. Costa

**Affiliations:** 1 Medicine Department, Federal University of Sergipe, Aracaju, Brazil; 2 Medicine Department Lagarto, Federal University of Sergipe, Lagarto, Brazil; 3 University Hospital Of The Federal University Of Sergipe, Federal University of Sergipe, Aracaju, Brazil; 4 Pharmacy Department Lagarto, Federal University of Sergipe, Lagarto, Brazil

**Keywords:** Mental disorders, Medical Education, mental health, Medical Students

## Abstract

**Introduction:**

Common Mental Disorders (CMD) are minor manifestations of depressive, anxious or somatoform symptoms, which do not fit the diagnostic criteria of the International Code of Diseases (ICD). In medical students, this panorama can generate even more repercussions given the complexity of the medical education process.

**Objectives:**

Estimate the prevalence and recognize associated factors of CMD among medical at the Federal University of Sergipe, Brazil.

**Methods:**

A cross-sectional study was performed with randomly selected students between April and June 2019. The Self Report Questionnaire (SRQ-20) were used, along with a questionnaire about socioeconomic and demographic characteristics, personal aspects and educational process, prepared by the authors and previously tested in a pilot study. Statistical evaluation of multiple variables was performed through backward stepwise logistic regression analysis.

**Results:**

The study included 80 students, equivalent to 22.59% of the total population of the studied Campus. There was an age average of 23.2 years (± 4.12), mostly female (52.5%) and single individuals (35%). The prevalence of CMD was 50% and an association was observed with the following factors: feeling of dissatisfaction with the course (p = 0.034); consider their own academic performance poor or regular (p = 0.12); lack of physical activity (p = 0.043); being anxious when not using a cell phone (p = 0.007); and the retraction pattern in the face of conflict situations in their interpersonal relationships (p = 0.025).

**Conclusions:**

Results suggest a high prevalence of CDM, associated mainly with the personal perspective about the educational process and personal habits.

